# What Is the Best Available Evidence for Using Homeopathy in Patients with Intellectual Disabilities?

**Published:** 2014-07-04

**Authors:** Farshad Shaddel, Marjan Ghazirad, Mark Bryant

**Affiliations:** 1 University of Oxford; 2 Dermot Rowe Library, Oxford Learning Disability, United Kingdom

**Keywords:** Homeopathy, Intellectual Disability, Mental Retardation, Learning Disability, Systematic Review

## Abstract

***Objective:*** The debate about the effectiveness of homeopathy hits the headlines from time to time. Reported evidences for the role of homeopathy in psychiatric illness relevant to people with intellectual disabilities are patchy and inconsistent. In this review we summarize the best available evidence for the use of homeopathy to treat the psychiatric disorders common in this population.

***Methods:*** Systematic literature review was conducted through February 2012 to July 2012 in AMED, CINHAL, BNI, EMBASE, MEDLINE, PSYCHINFO and GOOGLE SCHOLAR. In the next steps thirty eight homeopathic associations were contacted and a top-up literature search was done on Scopus and World of Science databases till March 2014. Twelve relevant clinical trials were identified and included in this study. The quality of each trial was assessed by the Oxford quality scoring system (Known as Jadad score) as well as subjective review by two reviewers independently (good versus poor).

***Findings*** ***:*** The largest body of evidence pertained to the use of homeopathy in the treatment of attention deficit hyperactivity disorder (ADHD). There is heterogeneity in the quality of trials and also the outcome of studies but overall our findings suggest some potential for using homeopathy in ADHD. Current evidences do not support the use of homeopathy for treatment of speech and language difficulties. There was only one trial concerning the use of homeopathy in Autistic Spectrum Disorder. This was of a poor quality and unable to provide any recommendation.

***Conclusion:*** Whilst acknowledging the risk of publication and language bias in our study, the currently available evidences are neither conclusive nor comprehensive enough to give us a clear picture for the use of homeopathy in patients with intellectual disabilities. There are large gaps in the body of evidence concerning the role of homeopathy in the treatment of common disorders in intellectual disability, such as autism, challenging behavior or developmental arrest in childhood.

## Introduction

The debate about the effectiveness of homeopathy hits the headlines from time to time. Homeopaths have always reported the effectiveness of homeopathy in treating many mantel disorders and provided lower levels of evidence for example, case presentation, case series and observational studies to support this. However, difficulty in tailoring a high quality blinded randomized clinical trial (RCT) to the individualized nature of homeopathic treatment, has been a key issue over recent decades^[^^1^^,^^2^^]^. A brief overview of the published literature indicates that hundreds of clinical trials have been conducted on the application of homeopathy to a variety of illnesses, but with inconclusive overall results^[^^3^^-^^7^^]^. There is an evident need for more systematic reviews in the field. 

 Likewise evidence for the role of homeopathy in psychiatric illness and specially those relevant to people with Intellectual Disabilities (ID) are patchy and inconsistent^[^^8^^]^. In this study we will try to summarize the best available evidence for the use of homeopathy to treat the psychiatric disorders common in this population.

## Subjects and Methods

The articles included in the review were selected as follows:

 (i) Seven databases (AMED, CINHAL, BNI, EMBASE, MEDLINE, GOOGLE SCHOLAR and PSYCHINFO) were searched for different keywords (27 different combinations) for articles published by August 1, 2012 over the period between February 2012 to August 2012. This stage produced 28 results.

 (ii) The abstract of these articles were reviewed manually by two authors (F.S. & M.G.). Sixteen papers considered relevant. The hard copy retrieved out of which eight did meet the inclusion criteria. Inclusion criteria were being a randomized clinical trial on the use of homeopathy in the management of common mental disorders in people with intellectual disability. These eight papers were deemed appropriate for a citation search using Google Scholar (which has a feature for following up the citations of each article by other papers). This search generated an additional two relevant studies. 

 (iii) As the next step a reference cross-check was conducted manually on all of the included papers – additional articles were not identified. 

 (iv) To reach the gray literature, thirty eight homeopathy associations around the world were contacted and asked for informing us about any relevant un-published, published in non-indexed journals and conference abstracts. This generated additional two relevant clinical trials. The full texts were retrieved.

 (v) And finally Scopus and World of Science

database were searched for similar keywords till March 2014 to see if any other trial has been published since our initial search in 2012 - additional articles were not identified

 (vi) All searches were confined to articles written in English

**Fig. 1:**
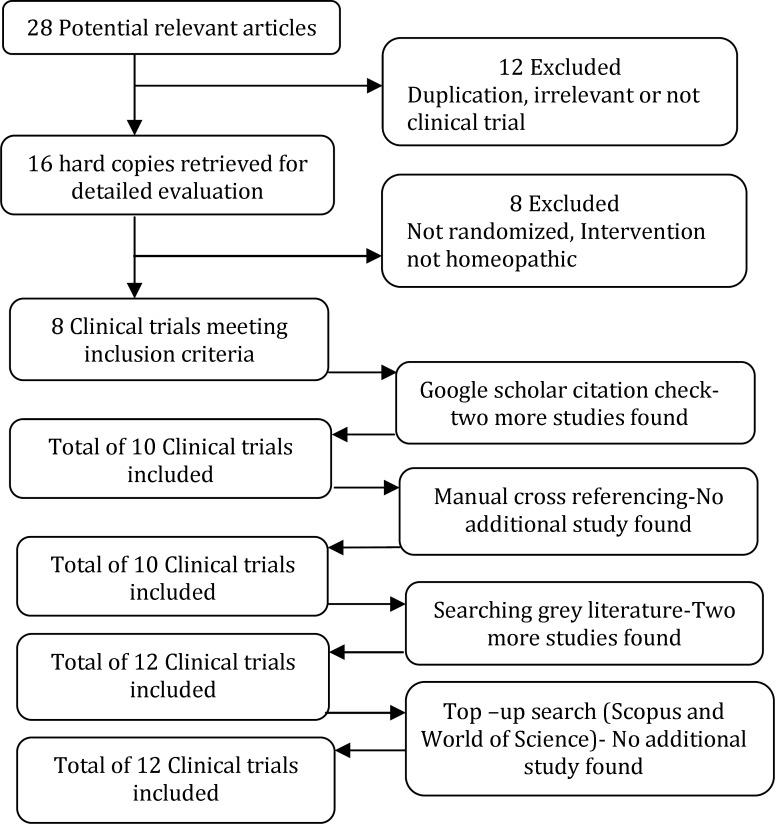
Flowchart of trial selection process

 The process of including studies is summarized in Fig 1. 

*Validity assessment: *The studies meeting the criteria were critically appraised. We used the Oxford quality scoring system (Known as the Jadad score) in addition to subjective independent reviews by two reviewers to assess the quality of included clinical trials. Reviewers were blinded to each others’ opinion to improve the reliability of appraisal. They divided the trials to two groups of good quality or poor quality ones.

 The Oxford quality scoring system is the most widely used tool for methodological assessment of the quality of a clinical trial^[^^9^^-^^11^^]^. It is a three-point questionnaire that forms the basis for a Jadad score. Each question is answered with either a yes or a no. Each yes scores a single point, each no zero points; there are no fractional points. The Jadad team states that they expect scoring any individual paper to take no longer than ten minutes. 

 The questions used to score papers which met the inclusion criteria were as follows:

1. Was the study described as randomized?
2. Was the study described as double blind?
3. Was there a description of withdrawals and dropouts?

To receive a point, an article should have described the number of withdrawals and dropouts in each study group, and the underlying reasons.

 Additional points were given if:

1. The method of randomization was described in the paper, and that method was appropriate.
2. The method of blinding was described, and it was appropriate.

Points were deducted if:

- The method of randomisation was described, but was inappropriate.
- The method of blinding was described, but was inappropriate.

Therefore a paper reporting a clinical trial could receive a Jadad score of between zero and five. For the purposes of our study, we considered a score of 3-5 as good quality and score of 0-2 as poor quality.

We exceptionally included findings of only one study without subjective critical appraisal because it has been already reviewed by another systemic review with a similar methodology^[^^12^^]^.

## Findings

Twelve studies met our inclusion criteria and were considered for critical appraisal. The qualities of included trials are summarized in Table 1. 

 The trials were on the use of homeopathy in management of the following mental disorders: Attention Deficit Hyperactivity Disorder (ADHD), Autism, Dyslexia and Speech & Social development in Cerebral Palsy (CP) patients. Most papers were authored or co-authored by homeopaths. In these twelve trials a total of 560 patients were studied for an average of 30 weeks, with a range of 4-156 weeks.

 There were eight clinical trials concerning the use of homeopathy for the treatment of ADHD with the total sample size of 385 patients. Three good quality trials^[^^13^^-^^15^^]^ with the total sample size of 187 patients and an average of 24 weeks follow up suggested positive outcomes for the use of homeopathy in ADHD whilst two other good quality trials^[^^16^^,^^17^^]^ with the total sample size of 63 patients and average follow up for 11 weeks, failed to identify decisive effectiveness for homeopathic treatment. 

 One of these studies^[^^16^^]^ compared a homeopathic complex (Quietude^®^) with “Advanced Brain Food” in the treatment of 20 patients for one month. This study reported very mixed findings with small improvements or deteriorations in some aspects of ADHD. In summary the study suggests the superiority of a special dietary supplement over homeopathy in the treatment of ADHD. The remaining two trials^[^^18^^-^^20^^]^ were of poor quality and therefore their findings were not considered as reliable.

 Dyslexia and other speech difficulties are common presentations in patients with learning disabilities. 

**Table 1:** Summary of trials related to use of homeopathy in patients with learning disabilities

**Primary author**	**Illness/Method**	**Trial**						**Quality**
		**n**	**OP**	**Outcome**	**Jadad Score**	**Reviewer 1**	**Reviewer 2**	
**Bull (2007) **	**Dyslexia/Sunflower**	70	16 w*	±	3	Good	Poor	Good
**Dhawale (Unpublished)**	**Dyslexia/Homeopathy**	69	3 y***	+	3	Poor	Poor	Poor
**Oberai (2013)**	**ADHD/Homeopathy**	61	1 y	+	4	Good	Good	Good
**Jacobs (2005)**	**ADHD/Homeopathy**	43	18 w	±	5	Good	Good	Good
**Sajedi (2007)**	**Speech and Social Development/ Homeopathy**	24	4 m**	-	5	Good	Good	Good
**Lottering (2006) **	**ADHD/Homeopathy vs Advanced Brain Food**	20	1 m	±	4	Good	Good	Good
**Robinson (2001)**	**Autism/Homeopathic Secretin**	12	12 w	-	1	Poor	Poor	Poor
**Lamont (1997)**	**ADHD/Homeopathy**	43	2 m	+	5	Good	Poor	Good
**Strauss (2000)**	**ADHD/Homeopathy**	20	2 m	+	2	NA	NA	Poor
**Frei (2007)**	**ADHD/Homeopathy**	83	12 m	+	1	Good	Poor	Poor
**Frei (2005)**	**ADHD/Homeopathy**	83	12 w	+	5	Good	Good	Good
**Frei (2001)**	**ADHD/Homeopathy**	115	6 m	Equal MPD****	1	poor	Poor	poor

* Week;

** Month;

*** Year;

**** Methylphenidate

There were three clinical trials^[^^21^^-^^23^^]^ on the use of homeopathy for speech difficulties (total of 163 patients and average 62 weeks follow up). Two good quality studies^[^^21^^,^^22^^]^ found no significant clinical improvement after using homeopathic treatment. One of them^[^^21^^]^ used sunflower therapy for the treatment of children with specific learning difficulties (Dyslexia). This study showed some small improvement in the self-esteem of children but no significant cognitive or literacy improvement. The other one^[^^22^^]^ added homeo-pathy to a rehabilitation program for a cerebral palsy patient with speech difficulties. This study could not identify any significant difference between the case and control group. The only study which suggested positive response on use of homeopathy in Dyslexia and Dysgraphia^[^^23^^]^ was of poor quality. 

 There was only one^[^^24^^] ^pilot trial with the sample size of 12 patients on the use of homeopathic preparation of Secretin for the treatment of autistic patients. This study suffers from significant methodological deficits and has a poor quality. Study reported no improvement in the patient.

## Discussion

Before any discussion on the findings of this review, it is important to acknowledge the limitations first. The main limitation of this systematic review is confinement to English language publications in indexed journals. This would impose the risk of language and publication bias. We tried to overcome this bias by writing to national homeopathic associations around the world and asking them to inform us about any un-published and non-English paper on use of homeopathy in people with ID. No study in other language was identified.

 Homeopathy in management of ADHD which is a common illness in patient with ID was the most studied area. Overall few good quality trials with longer follow up and bigger sample sizes have suggested some positive effects. The most cited study here, undertaken by Frei and colleagues on 83 ADHD patients, supports the effectiveness of homeopathy in the treatment of ADHD^[^^20^^]^. However, its validity and soundness of the findings have been challenged by other authors later on^[^^25^^]^. More high quality clinical trials are needed to facilitate a meta-analysis. 

 Our findings suggest that current evidences do not support the use of homeopathy for the management of speech difficulties.

 Surprisingly and despite widespread reports of using homeopathy for management of Autism in Medias and internet, there was only one clinical trial on this. The quality of trial was poor and therefore the findings could not be relied on. It is also worth mentioning there are some queries even regarding the effectiveness of intravenous Secretin therapy in Autistic patients as well^[^^26^^]^. Our conclusion is that currently there is no evidence in favor or against the effectiveness of homeopathy for the management of Autistic Spectrum Disorder.

 There are large gaps in the body of evidence for the use of homeopathy in the treatment of other common conditions associated with intellectual disability, such as epilepsy, challenging behavior and developmental arrest in childhood.

## Conclusion

Collectively the currently available evidence is neither conclusive nor comprehensive enough to give us a clear picture about the use of homeopathy in patients with intellectual disabilities. At least a few well designed clinical trials in each field are needed to be able to provide reasonable recommendations for the role of homeopathy in this population.
